# Optimizing a Protocol to Assess Immune Responses after SARS-CoV-2 Vaccination in Kidney-Transplanted Patients: In Vivo DTH Cutaneous Test as the Initial Screening Method

**DOI:** 10.3390/vaccines9111315

**Published:** 2021-11-12

**Authors:** Yvelise Barrios, Aurelio Rodriguez, Andrés Franco, Cristina Alava-Cruz, Domingo Marrero-Miranda, Lourdes Perez-Tamajon, Victor Matheu

**Affiliations:** 1 Department of Immunology, Main Hospital Universitario de Canarias, 38320 San Cristóbal de La Laguna, Spain; ybarpin@gobiernodecanarias.org (Y.B.); aframas@gobiernodecanarias.org (A.F.); 2 Department of Nephrology, Hospital Universitario de Canarias, 38320 San Cristóbal de La Laguna, Spain; arodherl@gobiernodecanarias.org (A.R.); dmarrero72@hotmail.com (D.M.-M.); mpertam@gobiernodecanarias.org (L.P.-T.); 3 Department of Allergy, Hospital Universitario de Canarias, 38320 San Cristóbal de La Laguna, Spain; calacru@gobiernodecanarias.org

**Keywords:** delayed-type hypersensitivity, DTH, skin test, SARS-CoV-2, vaccination, kidney transplant, immunosuppression, T-cell response

## Abstract

Previously, the delayed-type hypersensitivity (DTH) cutaneous test with the spike protein of SARS-CoV-2 has been shown to be a simple in vivo method to measure T-cell functionality after natural infection and in vaccinated individuals. Methods: Twenty-five kidney-transplanted recipients were immunized with two doses of the mRNA-based Pfizer–BioNTech COVID19 vaccine three weeks apart. Cell-immune response (CIR) was evaluated ten weeks later using an in vivo DTH skin test and in vitro with an interferon gamma release assay (IGRA). Humoral Immune Response (HIR) was determined by the measurement of specific IgG anti-S1 SARS-CoV-2. Results: Ten weeks after the second dose of the vaccine, 23 out of 25 transplanted patients had a positive DTH skin test, while in vitro CIR was considered positive in 20 patients. Unspecific stimulation was positive in all 25 patients, showing no T-cell defect. Seven out of twenty-five patients had a negative specific anti-spike IgG. CIR was positive in all immune-competent control patients. Conclusions: DTH is a useful, simple, and cheaper tool that can be used to assess cellular immune response, with an excellent correlation with the in vitro CIR. CIR assessment after vaccination in these immunocompromised patients is an excellent complement to HIR-based methods. This skin test could be used if classical in vitro methods cannot be applied.

## 1. Introduction

The measurement of the immune response against SARS-CoV-2 has been a hot topic since the emergence of the pandemic in 2020. During these months of the COVID-19 pandemic, a lot of research has been directed toward dissecting the humoral response. From these studies, it is now accepted that there are many reliable standard serological enzyme-linked immunoassays (ELISA) in the market, some of which even correlate uniformly with virus neutralization titers. However, in recent studies, some authors have seen impaired humoral immunity to the SARS-CoV-2 BNT162b2 vaccine in kidney transplant recipients and dialysis patients [1,2,3]. In addition, to investigate the role of T cells, many laboratories have started in vitro assays [4]. ELISA antibody methods and in vitro cellular tests require the extraction of a blood sample from the patient, which complicates the possibility of massive analysis in large populations. For these reasons, an alternative method that could be accomplished in such high-throughput investigations is needed. Cutaneous antigen-recall models allow the study of human memory responses in vivo. In this report, we show the use of the classical delayed-type hypersensitivity (DTH) cutaneous response to the intradermal injection of a recombinant protein representative of the SARS-CoV-2 virus to assess T-cell-mediated immune response in a group of kidney-transplanted patients.

## 2. Materials and Methods

### 2.1. Study Design

The patients underwent a medical consultation during July 2021. The results from 25 adults with immunosuppression for kidney transplantation were included. Demographic details were collected from all participants at the time of study. These patients were vaccinated with the BNT162b2 mRNA Pfizer vaccine via intramuscular administration. The vaccine administration schedule was carried out on days 0 and 21.

Each subject that intended to enter the study was given a written document called a “Patient Information Sheet”, which contained relevant and necessary information for the patient to decide whether they wanted to participate in the study. Treatment, communication, and transfer of the personal data of all participating subjects comply with the provisions of the law on the protection of personal data. A group consisting of twenty-five immunocompetent individuals of the same age and sex was used as a control group. The protocol was approved by the ethical committee of the Hospital (CHUC_2020_92 and CHUC_2021_04) and was conducted in accordance with the requirements expressed in law.

### 2.2. Specific IgG to SARS-CoV-2 Spike

Blood samples were collected from the participants ten weeks after their second dose of vaccine, and every single serum was frozen at the Immunology laboratory at −20 °C until use. A commercial ELISA to detect specific antibodies immunoglobulin (Ig) G (IgG) for the S1 protein of SARS-CoV-2 was used in accordance with the manufacturer’s instructions (SARS-CoV-2 IgA and IgG immunoassay, *Euroimmun*, Lübeck, Germany). All determinations were made with serum samples diluted by 1:100 with paired samples and were analyzed in the same assay. The results were expressed in mg/mL (results under 5 mg/mL were considered negative). 

### 2.3. T-Cell Response by Specific DTH Cutaneous Test

On the same day as the blood collection [5], after oral and written informed consent and after sterilization with alcohol in the volar part of the arm, a reconstituted lyophilized SARS-CoV-2 recombinant protein of the receptor binding domain (RBD, 25 μL (final concentration of 0.1 mg/mL)) similar to the dose normally used in the tuberculin test [6,7] was administered as the intradermal test (IDT) puncture, with an immediate reading after 15 min [6,7]. The protocol was performed according to usual clinical practice in the area of diagnostic techniques of the Allergy Service [5]. Intradermal tests were carried out ten weeks after vaccination and were not performed in patients with a history of grade II or higher anaphylaxis.

The patients were instructed to take a photograph of the part of the arm with the puncture at agreed times (6 h, 12 h, 24 h, 36 h, 48 h, and 72 h after injection) and to add a measuring ruler next to it for reference [8]. They were given a telephone number for assistance 24 h a day for consultation and evaluation if necessary. DTH readings in mm were obtained by a blind reading by 3 different researchers outside the study based on the results obtained from the photographs.

### 2.4. T-Cell Response by In Vitro Stimulation with SARS-CoV-2 Spike Protein

An automated commercial in vitro diagnostic method that measures a component of cell-mediated immune reactivity to the S1 protein of SARS-CoV-2 was performed (SARS-CoV-2 Interferon Gamma Release Assay (IGRA), Euroimmun, Lübeck, Germany). If a patient has been in contact with the S1 protein of SARS-CoV-2, their white blood cells will release IFN-gamma in response to contact with the antigen. The assay was performed using fresh whole blood collected from the study subjects using the following method. Briefly, human lithium–heparin whole blood was obtained from each patient and 500 uL was distributed and mixed on each tube of one set of stimulation tubes (#1: blank, #2: S1 domain from the spike protein, #3: mitogen causing unspecific IFN-gamma secretion from T cells). Incubation at 37 °C for 16 h was performed to promote the release of interferon gamma by T cells in response to contact with the antigen. Finally, individual supernatants of these stimulated samples were collected, and an IFN-gamma ELISA (Euroimmun) was performed following manufacturer’s instructions. Calibrated standards were included and expressed in International Units per milliliter (IU/mL).

### 2.5. Statistics Analysis

The data were collected and analyzed by the authors. Differences between the distributions of continuous variables were evaluated using the 2-tailed Mann–Whitney U test. Correlation analyses were carried out using Pearson’s correlation. Differences were considered statistically significant when the p was less than 0.5.

## 3. Results

### 3.1. Demographic Data

Twenty-five individuals (7 female/18 male) with a mean age of 53.3 y-o were analyzed. The average time since transplant was 16 years. The individual immune-suppression scheme for each of the subjects is described in the following table (Table 1). The control group consisted of 17 women and 8 men with a mean age of 52 years and a median of 51 years and who had been vaccinated following the same vaccination schedule against COVID-19, with two doses of the Pfizer vaccine with an interval between doses of 3 weeks.

### 3.2. Specific Anti-RBD IgG

**Figure 1:**
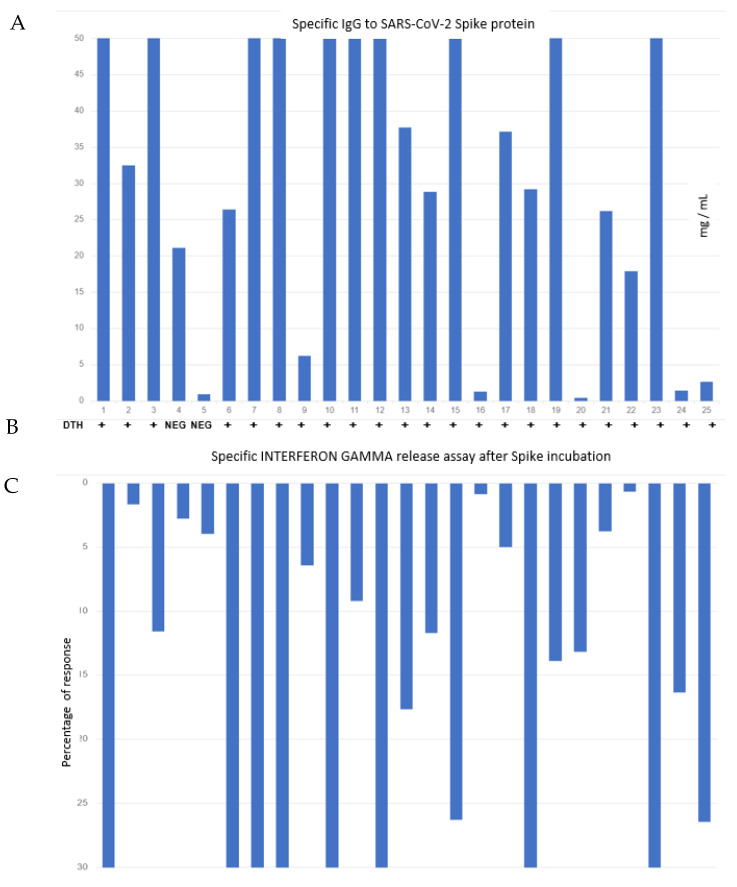
(**A**) Levels of specific antibody IgG against spike protein in mg/mL. Levels above 5 mg/mL are considered as positive serology. (**B**) Table with results of DTH (qualitative) in every single patient. (**C**) Percentage of response with respect to the levels of interferon gamma present in the supernatant after the incubation of whole blood from each patient with the SARS-spike protein. Levels beyond 5% are considered positive.

Levels of specific IgG anti-spike are shown in Figure 1. Five individuals (#5, #16, #20, #24 and #25) had specific IgG levels below the detection limit considered positive (5 mg/mL). Of the remaining 20 individuals who had positive specific IgG levels, 19 had high levels.

### 3.3. IFN-Gamma Levels after Incubation of T-Cells with Spike RBD

Measurements of IFN-gamma levels after the specific stimulation of T cells with the spike antigen of SARS-CoV-2 are shown in Figure 1C as percentages of response (compared to stimulation with an unspecific stimulus, in which we determined unspecific T-cell activity). Seven individuals (#2, #4, #5, #16, #17, #21 and #22) had levels below the limit considered positive (under 5% of response). Of the remaining 18 individuals who had positive IFN-gamma levels, 17 were above 10% of the maximum reached by the unspecific stimulation tube. Statistical analysis showed a Pearson’s correlation coefficient of r = 0.491 between IGRA and IgG titers (*p* < 0.01) and Pearson’s r = 0.407 between DTH at 24 h and IGRA (*p* < 0.05). 

### 3.4. RBD Recombinant Protein Skin Test (DTH)

**Figure 2:**
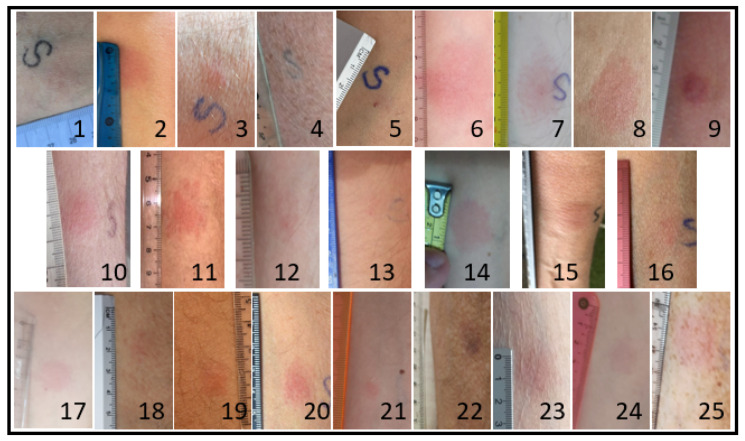
Samples of the photographs of the response in the arm of each of the 25 patients after 48 h of the DTH test.

**Figure 3:**
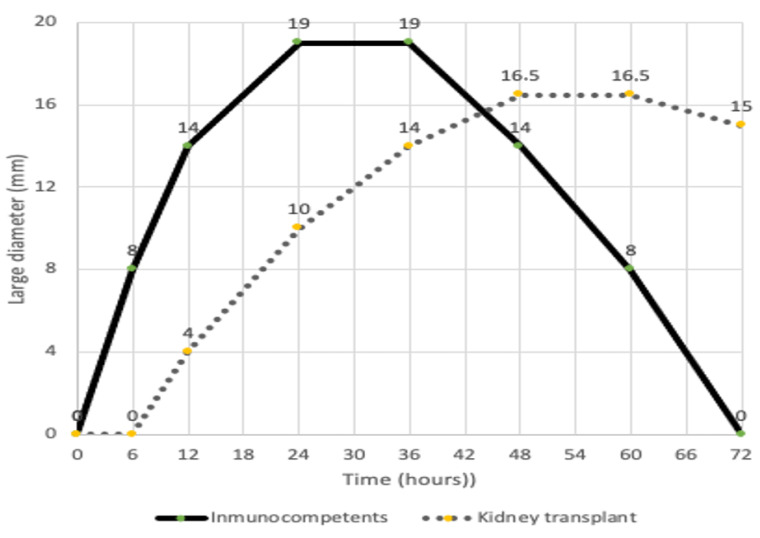
Kinetics of the median diameter in mm based on the cutaneous DTH response of kidney-transplanted patients (dashed line; n = 23) after intradermal test and compared with the control group of immuno-competent individuals (solid line; n = 25).

Twenty-three out of twenty-five patients tested positive in the RBD skin test (Figure 2). The kinetics of the positive cutaneous tests was (median) 4 mm (12 h), 10 mm (24 h), 14 mm (36 h), 15 mm (48 h), and 15 mm (72 h) after the intradermal injection. Statistical analysis showed a Pearson’s correlation coefficient of r = 0.425 (*p* < 0.05) between DTH 48 h and IGRA but a r = 0.197 (*p* = 0.346) between DTH 48 h and IgG titers. The kinetics of the skin test in kidney transplant patients were slightly different compared to immunocompetent individuals (Figure 3), and we found statistically significant differences at the 12 h, 24 h, and 72 h timepoints. 

## 4. Discussion

The main objective of immunosuppressive treatment in kidney transplant patients is aimed to control the response of recipient T lymphocytes against the donor allogenic antigens. However, in this study, we found that despite immunosuppressive treatment, transplanted patients present an acceptable T response to the SARS-CoV-2 vaccine, evaluating both the production of interferon-gamma “in vitro” and the skin response. One of the reasons for the superior results using DTH compared with the IGRA assay might be because a more functional presentation of the antigen is achieved through the in vivo uptake and processing of the antigen by professional APC residents in the skin. The immune status of these individuals could be better represented by an in vivo approach.

Most of the published works show a low percentage of response to the vaccine in transplanted patients [9,10,11,12]. However, in this study, we found an important response after two doses of SARS-CoV-2 messenger RNA (mRNA) vaccine. We showed that the SARS-CoV-2 vaccine has a delayed response in immunocompromised patients, reaching a maximum several weeks after that of immunocompetent individuals. In some published references, the evaluation of the immune response was carried out four weeks after the administration of the second dose of vaccine. This period is adequate in the normal population, but in immunocompromised patients, it may be insufficient. Recently, in a published trial on the response to a third dose of RNA-COVID vaccine in transplant patients versus placebo, the authors found an estimable increase in the anti-RBD IgG antibody titers in the placebo group [12]. A possible explanation for this increase in the titer of antibodies in the patients who were administered the placebo could be that they had not yet achieved the adequate response after the second dose of the vaccine, reaching maximum values a few weeks later, coinciding with the administration of the placebo. In our study, the assessment of the response to two doses of the vaccine in transplant patients was assessed ten weeks after the administration of the second dose, which could justify our finding of a higher response rate in both the IgG antibody titers and also in the anti-RBD in vitro production of gamma interferon. Other studies [13,14] have studied immune response much earlier, usually during the second week after vaccine administration. 

This study has certain limitations. First, the number of transplanted patients studied is low, since our objective was to gain preliminary data about the confirmation of the skin test in these group of patients. A second limitation is the intrinsic low sensitivity of any study of T lymphocytes in immunosuppressed patients, although this effects in vitro and in vivo studies [15]. Another limitation is the difficulty of having a homogeneous reading of the DTH since our test is not yet standardized, although this drawback has been partially overcome by using the same group of experts interpreting the results. However, a broader and multicenter study would be necessary to be able to standardize the reading of the test. In the future, the protocol of communication with patients needs to be improved to enhance the quality of the photographs and their submission of these images in the stipulated time through a mobile phone. 

A third dose, as promoted by some authors [16], should undergo a previous immune study. After the excellent results obtained with the in vivo DTH skin test in different clinical scenarios, we believe that, at least in some population groups with a certain degree of immunosuppression, it would be suitable to include this study prior to considering a booster of their conventional vaccination regimen. In the case of large populations, the specific in vivo study via DTH cutaneous tests would provide a reliable, safe, low-cost, and efficient approach.

## Figures and Tables

**Table 1:** Demographic data of all patients, including the year of transplantation, immunosuppressive agent, and doses in mg. M: male; F: female; YearTX: year of kidney transplant; Cicl: ciclosporine; AZ: azathioprine; Ev: everolimus; S: sirolimus.

	Age	Gender	YearTx	Immunosuppressive Agent (in mg)				
				Prednisone	Tacrolimus	Mycophenolate	Cicl	Other
1	50	M	1986	5	2	-	-	-
2	35	M	2000	5	3	750	-	-
3	50	M	1991	5	1.5	-	-	-
4	48	M	2006	-	1.5	1500	-	-
5	62	M	2004	2.5	3.5	1000	-	-
6	54	F	1999	5	3.5	750	-	-
7	49	M	1999	-	-	750	100	-
8	57	F	1990	2.5	-	1500	100	-
9	64	M	1993	1.25	3.5	720	-	-
10	51	M	1987	-	-	-	-	AZ 50
11	44	M	2000	2.5	-	1000	-	-
12	54	M	1990	5	-	-	100	-
13	50	M	1999	7.5	-	1500	-	-
14	31	F	2002	3.5	-	750	-	-
15	68	F	1991	2.5	2	1000	-	-
16	79	M	2001	5	7	750	-	-
17	46	F	2001	5	12.5	360	-	-
18	65	M	2002	2.5	2.5	-	-	Ev 1.5
19	43	M	2002	-	-	500	-	S 0.5
20	54	M	1986	-	4	1000	-	-
21	52	M	2002	-	3	750	-	-
22	72	M	1991	5	3	1500	-	-
23	68	F	2000	-	-	360	200	-
24	35	F	1999	-	5	1000	-	-
25	53	M	1991	5	-	1000	-	Ev 2

## Data Availability

Data could be provided upon request.
